# Sex-specific immune-inflammatory markers and lipoprotein profile in patients with anhedonia with unipolar and bipolar depression

**DOI:** 10.1186/s12888-023-05378-4

**Published:** 2023-11-27

**Authors:** Shengjuan Lin, Rongxun Liu, Zhongguo Zhang, Fengyi Liu, Shisen Qin, Yange Wei, Fei Wang

**Affiliations:** 1 Early Intervention Unit, Department of Psychiatry, The Affiliated Brain Hospital of Nanjing Medical University, 264 Guangzhou Street, Nanjing, Jiangsu 210029 China; 2 Functional Brain Imaging Institute, Nanjing Medical University, Nanjing, China; 3 School of Psychology, Xinxiang Medical University, Xinxiang, Henan 453002 China; 4 The Fourth People’s Hospital of Yancheng, Yancheng, China; 5 School of Public Health, Xinxiang Medical University, Xinxiang, China; 6 Department of Early Intervention, Henan Mental Hospital, The Second Affiliated Hospital of Xinxiang Medical University, Xinxiang, Henan 453002 China

**Keywords:** Anhedonia, Depression, Immune-inflammatory markers, Lipoprotein profile, Sex

## Abstract

**Background:**

Anhedonia is a core symptom in patients with unipolar and bipolar depression. However, sex-specific markers reflecting biological heterogeneity are lacking. Emerging evidence suggests that sex differences in immune-inflammatory markers and lipoprotein profiles are associated with anhedonia.

**Methods:**

The demographic and clinical data, immune-inflammatory markers (CD3, CD4, and CD8), and lipoprotein profiles [TC, TG, LDL-C, HDL-C, lipoprotein(a) Lp (a)] of 227 patients with unipolar and bipolar depression were collected. The Hamilton Depression Rating Scale (HAMD) and Snaith-Hamilton Pleasure Scale (SHAPS) were used to assess depression and anhedonia symptoms. Data were analyzed using ANOVA, logistic regression, and receiver operating characteristic curves.

**Results:**

Male patients in the anhedonia group had higher levels of CD3, CD4, and CD8, and lower levels of Lp (a) than the non-anhedonia group, while no significant difference was identified in female patients with and without anhedonia. Logistic regression analysis showed that CD3, CD4, CD8, and Lp (a) levels were associated with anhedonia in male patients. Furthermore, the combination of CD3, CD4, CD8, and Lp (a) had the strongest predictive value for distinguishing anhedonia in male patients than individual parameters.

**Conclusions:**

We identified sex-specific associations between immune-inflammatory markers, lipoprotein profiles, and anhedonia in patients with unipolar and bipolar depression. The combination of CD3, CD4, CD8, and Lp (a) might be a possible biomarker for identifying anhedonia in male patients with unipolar and bipolar depression.

**Supplementary Information:**

The online version contains supplementary material available at 10.1186/s12888-023-05378-4.

## Background

Major depressive disorder and bipolar depression are chronic, debilitating mental disorders characterized by repeated episodes of depression, affecting up to 16.1 million people worldwide [1]. A large-scale psychiatric epidemiological survey in China reported that the lifetime prevalence of depressive disorders was 3.4% [2]. As the core symptom of both unipolar and bipolar depression, anhedonia is defined as a reduced capacity for pleasure or decreased motivation for rewards [3]. Up to 70% of patients with unipolar depression [4] and 52% of patients with bipolar depression [5] experience clinical anhedonia symptoms. Importantly, anhedonia patients in depression tend to have poorer treatment outcomes, higher functional impact, and suicide relative to unanhedonia patients [6–10]. Specifically, the associations between anhedonia, poorer disease prognosis, and unsatisfactory curative outcomes have been reported [11]. In a large sample (N = 1,570) of patients with major depression, Vinckier et al. showed that anhedonia was the strongest predictor of improvement in psychosocial functioning [12]. A total of 20 studies and 11,212 individuals indicated that anhedonia may be a core risk factor for suicidal ideation and behaviours [13]. Hence, an unmet clinical need remains to develop specific treatment strategies for patients with anhedonia.

As a prominent symptom of unipolar and bipolar depression, evaluation of anhedonia is mainly based on clinician-assessed or self-reported measures, such as Hamilton Depression Scale (HAMD), Snaith-Hamilton Pleasure Scale (SHAPS), and Temporal Experience of Pleasure Scale (TEPS) [14, 15]. SHAPS is a reliable and valid tool for clinician assessment of hedonic capacity in unipolar and bipolar depression [9]. It is a 14-item questionnaire covering four domains of hedonic experience: interest/ pastimes, social interaction, sensory experience, and food/drink [10]. TEPS is a well-validated self-report scale that quantifies both anticipatory and consummatory anhedonia [15, 16]. Anticipatory anhedonia is associated with emotional state in anticipation of future activities, while consummatory anhedonia reflects momentary state while experiencing pleasurable events [17, 18]. Souery et al. demonstrated more severe anhedonia in patients with unipolar depression than in bipolar depression [19], while Diler et al. reported contradictory findings [20]. One study showed differences in anticipatory anhedonia [21], another recent study identified differences in consummatory anhedonia [22] between unipolar and bipolar depression. Findings from these studies comparing anhedonia severity in patients with unipolar and bipolar depression are inconsistent [9].The fundamental reason for this is the lack of objective measures and mechanistic understanding of anhedonia. It is therefore essential to identify molecular biomarkers and determine the underlying mechanisms of anhedonia to develop more effective therapeutic strategies.

Compelling evidence suggests that sex-related factors play a role in the pathophysiology of anhedonia. According to the latest cross-sectional epidemiological study of the prevalence of mental disorders in China (32,552 respondents), the weighted 12-month prevalence of depressive disorders was 4.2% in female and 3.0% in male participants [2]. In animal models, Chronic Unpredictable Mild Stress (CUMS) male rats present depression-like symptoms, including anhedonia [23]. Bennett [24] appears to be the first to indicate that depressed male patients may have higher levels of anhedonia. Male patients (37.2% of the sample) had an older age at the onset of the first experience of depressive symptoms than female patients. Both sexes present with depressive symptoms consistent with alcohol and drug abuse [25]. Clinical studies have demonstrated that male patients with depression have worse response to selective serotonin reuptake inhibitors compared to female patients [26]. However, sex differences in anhedonia have received relatively little attention. Consequently, identification of sex-specific biomarkers that reflect the intrinsic biological behavior of anhedonia is urgently needed.

Understanding the biological underpinnings of sex differences in unipolar and bipolar depression may provide insights into the mechanisms of anhedonia [27]. As an immune response of the central nervous system, neuroinflammation has been implicated in the pathophysiology of anhedonia [6]. It can be activated by infectious agents, trauma, stress, aging, environmental toxins, and ischemia [28]. Numerous studies, including meta-analyses, have reported increased peripheral and central immune-inflammatory markers, such as T lymphocytes (CD3, CD4, and CD8), C-reactive protein, and pro-inflammatory cytokines in unipolar and bipolar depression [29–31]. Elevated inflammatory markers (namely, CD3, CD4, and CD8) have been previously associated with anhedonia [32, 33]. Meanwhile, neuroinflammation may arise from risk factors, including lipoprotein dysfunction, obesity, lifestyle factors, and genetics [6]. The lipoprotein profile, including total cholesterol (TC), triglycerides (TG), low-density lipoproteins (LDL), high-density lipoproteins (HDL), and lipoprotein(a) [Lp (a)] plays a crucial role in immune activation and accelerate the course of depression [34, 35]. Accumulating evidence from animal models and clinical studies has shown some abnormalities in immune responses, such as changes in immune-inflammatory markers and lipoprotein profile-linked anhedonia [36, 37]. Sex-specific differences in the association between immune-inflammatory markers, lipoprotein profile, and anhedonia may exist [35, 38]. The past few study combined immune-inflammatory markers and lipoprotein profiles to explore their roles as potential biomarkers of anhedonia in unipolar and bipolar depression according to sex. So far, no biomarker-based diagnostic panel is available for anhedonia.

Herein, we assess immune-inflammatory markers and lipoprotein profiles of male and female patients with anhedonia with unipolar and bipolar depression. This study had three main objectives: (1) to investigate whether female and male patients with anhedonia have distinct sex-specific serum biomarkers; (2) to evaluate the sex-specific association between immune-inflammatory markers, lipoprotein profile, and anhedonia in unipolar and bipolar depression; and (3) to estimate the performance of immune-inflammatory markers and lipoprotein profiles in identifying anhedonia in patients with unipolar and bipolar depression.

## Methods

### Participant

A total of 227 patients with unipolar and bipolar depression were recruited from the Department of Psychiatry of the Affiliated Nanjing Brain Hospital, Nanjing Medical University. Each patient was interviewed by two experienced psychiatrists using the Structured Clinical Interview for DSM-IV-TR-Patient Edition (SCID-P) and was finally diagnosed with unipolar or bipolar depression according to the DSM-IV. Patients were included only when the diagnosis was consistent between the two psychiatrists. The exclusion criteria for all participants were drug / alcohol abuse or dependence, concomitant major medical disorders, severe immune index/infectious diseases, or neurological disorders.

## Clinical assessment

Overall symptom severity was assessed using the 17-item Hamilton Depression Scale (HAMD-17) [39], Hamilton Anxiety Rating Scale (HAMA) [40], and Young Mania Rating Scale (YMRS) [41]. Self-report measures of hedonic experience included SHAPS and TEPS in this study. We first used the SHAPS to assess the presence of anhedonia. SHAPS items are scored 0 or 1 (definitely agree or agree = 0; disagree or definitely disagree = 1). We followed the original algorithm and SHAPS cut-off level (< 3 and ≥ 3 of normal / abnormal) for anhedonia by Snaith et al. [11]. All patients were divided into 2 subgroups, 126 subjects scoring ≥ 3 on SHAPS were regarded as anhedonic group, while 101 subjects scoring < 3 were non-anhedonic group. Then, anhedonia severity was assessed using the TEPS, a 18-item self-report questionnaire designed to measure anticipatory (TEPS-ANT, score range 10 to 60) and consummatory (TEPS-CON, score range 8 to 48) anhedonia [15, 16]. Lower TEPS scores represent greater levels of anhedonia [42].

### Plasma sample preparation and detection

Fasting blood samples (5 ml) were collected from the forearm veins between 8 am and 10 am after the participants had fasted overnight. Blood samples were then clotted at room temperature for 40 min and centrifuged at 3000 rpm for 10 min. Serum samples were separated before use, aliquoted, and stored in a refrigerator at − 80℃.

Immune-inflammatory markers, including CD3, CD4, and CD8, were examined by flow cytometry (BD FACS Calibur) using commercially available flow cytofluorometric (FCM) kits (Mindray, Shenzhen, China) (normal reference ranges: CD3, 770–2860µL; CD4, 414–1440µL; CD8, 238–1250µL). The lipoprotein profile, which includes TC, TG, LDL-C, and HDL-C levels, was assessed by enzymatic colorimetry using commercially available kits (Zhongsheng Beikong, Beijing, China) (normal reference ranges: TC, 2.8–5.18 mmol/L; TG, 0.28–1.7 mmol/L, LDL-C, 1.56–3.37 mmol/L; HDL-C, 1.04–2.2 mmol/L). Lp(a) was quantified using an immunoturbidimetric method (Desai, Shanghai, China). The (local reference range for normal Lp (a) level is 0-300 mg/L).

### Statistical analysis

All analyses were performed using Statistical Package for the Social Sciences for Windows, Version 22.0. First, patients were divided by sex: ’female’ and ‘male.’ The samples in each group were then subdivided into two subgroups: ‘anhedonia’ and ‘non-anhedonia.’ The Shapiro-Wilk test was used to test for normality of continuous variables. Data presented as mean and standard deviation (SD) were analyzed using Student’s t-test. Sex-related differences were analyzed with two-way analysis of variance (ANOVA, repeated measures within patients based on the factor of diagnosis, i.e., non-anhedonia vs. anhedonia; between patients based on factor of sex, i.e., female vs. male; and diagnosis × sex interaction) followed by the Bonferroni post hoc test. Second, we used multivariate logistic regression analysis to determine the factors associated with anhedonia in patients with unipolar and bipolar depression. Odds ratios (ORs) and corresponding 95% confidence intervals (CI) were estimated using logistic regression analysis. Third, we plotted receiver operating characteristic (ROC) curves and estimated the area under the curve (AUC) to further evaluate the performance of immune-inflammatory markers and lipoprotein profiles in discriminating anhedonia in patients with unipolar and bipolar depression. The cutoff values of each biomarker and the combined biomarkers were established from the analysis of ROC curves to achieve maximum specificity and sensitivity. All statistical tests were two-tailed, and a *P*-value of < 0.05 was considered statistically significant.

## Results

### Characteristics of study participants

In total (N = 227), 163 (71.8%) of the participants were female and 64 (28.2%) were male. As depicted in Table S1, there were no significant sex differences in age, BMI, HAMA total score, YMRS total score, TEPS total score, TEPS anticipatory score, and consummatory score (all *P* > 0.05). The total HAMD scores were significantly higher in female than male patients (*P* < 0.05).

Among female patients with unipolar (N = 79) and bipolar depression (N = 84), there were 91 and 72 patients in the anhedonia and non-anhedonia groups, respectively (Table S2). There were no significant differences in age, BMI, or YMRS score between the groups (*P* > 0.05). The anhedonia group demonstrated higher total HAMD and HAMA scores and lower total TEPS and TEPS anticipatory and consummatory scores (*P* < 0.001, Table 1).

**Table 1:** Demographic and clinical characteristics of patients with unipolar and bipolar depression

Characteristic	Male (N = 64)				Female (N = 163)			
	Non-anhedonia (N = 29)	Anhedonia (N = 35)	t	*P*	Non-anhedonia (N = 72)	Anhedonia (N = 91)	t	*P*
Age (years)	15.41 ± 1.43	15.74 ± 2.27	0.682	0.498	15.90 ± 2.21	15.29 ± 2.06	1.836	0.068
BMI (kg/m^2^)	25.61 ± 8.18	23.63 ± 6.69	-1.016	0.314	22.32 ± 6.37	23.36 ± 7.76	-0.859	0.392
HAMA total score	20.53 ± 9.08	23.14 ± 8.21	1.195	0.237	22.36 ± 7.80	24.88 ± 7.38	-2.097	0.038*
HAMD total score	19.78 ± 7.41	23.40 ± 6.36	2.068	0.043*	21.96 ± 6.85	25.39 ± 5.85	-3.432	0.001***
YMRS total score	9.33 ± 5.92	9.37 ± 6.19	0.025	0.980	9.86 ± 6.31	8.86 ± 6.39	0.995	0.321
TEPS total score	76.97 ± 17.04	57.09 ± 13.57	-5.197	<0.001***	78.47 ± 13.61	56.37 ± 11.87	9.199	<0.001***
TEPS ANT score	42.52 ± 10.04	32.54 ± 8.03	-4.415	<0.001***	43.42 ± 7.63	31.56 ± 6.97	10.144	<0.001***
TEPS CON score	34.45 ± 8.27	24.54 ± 6.71	-5.294	<0.001***	35.05 ± 6.81	23.01 ± 5.87	11.848	<0.001***

Abbreviations: BMI, body mass index; HAMA, Hamilton Anxiety Rating Scale; HAMD-17, Hamilton Depression Rating Scale-17; YMRS, Young Mania Rating Scale; TEPS, Temporal Experience of Pleasure Scale; TEPS ANT, TEPS anticipatory anhedonia; TEPS CON, TEPS consummatory anhedonia

* *P* < 0.05; ** *P* < 0.01; *** *P* < 0.001

Among male patients with unipolar (N = 29) and bipolar depression (N = 35), there were 35 patients in the anhedonia group and 29 in the non-anhedonia group (Table S2). There were no significant differences in age, BMI, HAMA, or YMRS scores between the anhedonia and non-anhedonia groups (*P* > 0.05). Compared to the non-anhedonia group, the anhedonia group showed significantly higher HAMD scores and lower total TEPS and TEPS anticipatory and consummatory scores (*P* < 0.001, Table 1).

### Sex-specific immune-inflammatory markers and lipoprotein profile of patients with anhedonia

**Fig. 1:**
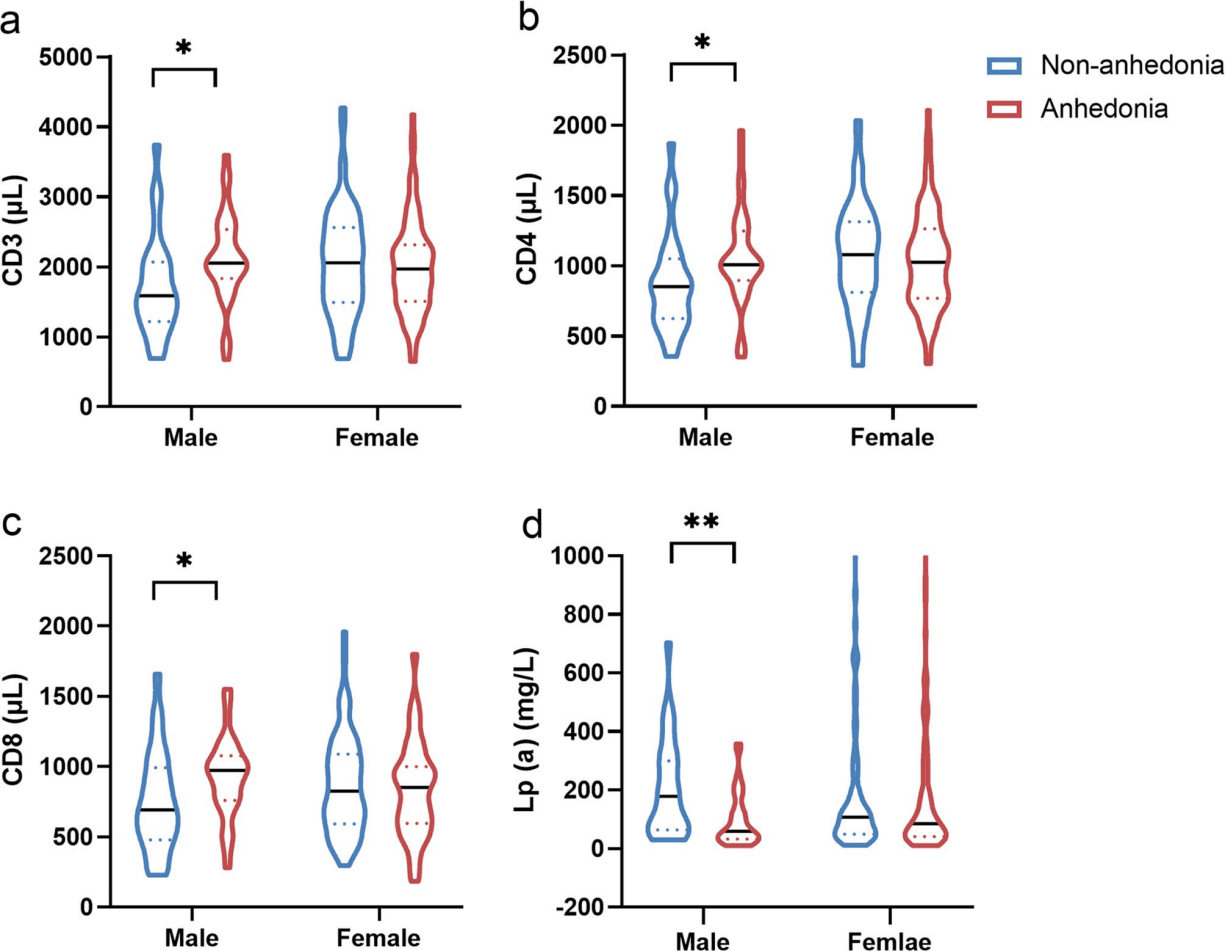
Plasma CD3, CD4, CD8 and Lp (a) levels between male and female or anhedonia and non-anhedonia patients with unipolar and bipolar depression. Plasma levels of CD3 (**a**, *t* = -2.354, *P* = 0.022), CD4 (**b**, *t* = -2.213, *P* = 0.031), CD8 (**c**, *t* = -2.142, *P* = 0.038) and Lp (a) (**d**, *t* = -3.013, *P* = 0.009) in the anhedonia group were significantly higher than the non-anhedonia group in male patients with unipolar and bipolar depression. ** *P* ≤ 0.01; * *P* ≤ 0.05. Abbreviation: Lp (a), lipoprotein(a)

As shown in Table 3; Fig. 1, two-way ANOVA demonstrated a significant sex interaction effect for CD3 (F = 4.675, *P* = 0.031), CD4 (F = 4.199, *P* = 0.042), CD8 (F = 4.375, *P* = 0.038), and Lp (a) (F = 7.051, *P* = 0.009). Post-hoc analysis showed significant effects in male patients, whereas female patients did not show statistically significant differences. Among male participants, patients with anhedonia showed significantly higher CD3, CD4, and CD8 levels and lower Lp (a) levels. Thus, we performed all subsequent analyses only in male participants (Fig. 1; Table 2).

**Table 2:** Immune-inflammatory markers and lipoprotein profile in patients with anhedonia and non-anhedonia

Characteristic	Male (N = 64)				Female (N = 163)			
	Non-anhedonia (N = 29)	Anhedonia (N = 35)	t	*P*	Non-anhedonia (N = 72)	Anhedonia (N = 91)	t	*P*
CD3 (µL)^a^	1731.86 ± 713.60	2128.69 ± 634.27	-2.354	0.022*	2056.72 ± 745.89	2006.84 ± 673.49	0.448	0.655
CD4 (µL)^b^	872.14 ± 349.46	1046.91 ± 344.94	-2.213	0.031*	1063.67 ± 378.65	1038.95 ± 348.41	0.433	0.666
CD8 (µL)^c^	750.48 ± 354.22	920.91 ± 282.34	-2.412	0.036*	858.94 ± 362.21	833.82 ± 333.92	0.459	0.647
TC (mmol/L)	4.01 ± 0.82	3.98 ± 1.10	0.130	0.897	4.15 ± 0.79	4.08 ± 0.98	0.492	0.623
TG (mmol/L)	1.28 ± 0.70	1.18 ± 059	0.600	0.551	1.12 ± 0.81	0.95 ± 0.50	1.572	0.118
LDL (mmol/L)	2.13 ± 0.59	2.13 ± 0.83	0.135	0.979	2.11 ± 0.60	2.13 ± 0.67	-0.140	0.889
HDL (mmol/L)	1.21 ± 0.21	1.15 ± 0.20	1.154	0.253	1.32 ± 0.24	1.33 ± 0.37	-0.239	0.812
Lp (a) (mg/L)^d^	205.79 ± 169.17	96.88 ± 91.57	3.227	0.002**	180.81 ± 217.16	194.39 ± 282.47	-0.332	0.740

Abbreviations: TC, total cholesterol; TG, triglycerides; LDL, low density lipoproteins; HDL, high density lipoproteins; Lp (a), lipoprotein(a)

* *P* < 0.05; ** *P* < 0.01; *** *P* < 0.001

^a^Group (anhedonia or non-anhedonia)-by-sex (male or female) interaction was observed for CD3, *P* = 0.031 (two-factor ANOVA)

^b^Group (anhedonia or non-anhedonia)-by-sex (male or female) interaction was observed for CD4, *P* = 0.042 (two-factor ANOVA)

^c^Group (anhedonia or non-anhedonia)-by-sex (male or female) interaction was observed for CD8, *P* = 0.038 (two-factor ANOVA)

^d^Group (anhedonia or non-anhedonia)-by-sex (male of female) interaction was observed for Lp (a), *P* = 0.009 (two-factor ANOVA)

### Sex-specific association between immune-inflammatory markers, lipoprotein profile, and anhedonia

The multivariate binary logistic regression model showed that CD3, CD4, CD8, and Lp (a) were independent predictors of anhedonia in male patients with unipolar and bipolar depression (Table 3).

**Table 3:** Binary logistic regression analyses of anhedonia in male patients with unipolar and bipolar depression

Varibales	β	SE	Wald	Exp (β)	95% CI	*P*
Age	0.017	0.182	0.009	1.018	0.713, 1.453	0.923
CD3	0.043	0.020	4.480	1.043	1.003, 1.085	0.034*
CD4	-0.043	0.021	4.103	0.958	0.920, 0.999	0.043*
CD8	-0.046	0.022	4.493	0.955	0.915, 0.997	0.034*
Lp (a)	-0.009	0.003	7.425	0.991	0.985, 0.998	0.006**

Abbreviation: Lp (a), lipoprotein(a)

* *P* < 0.05; ** *P* < 0.01; *** *P* < 0.001

### ROC curve analysis of immune-inflammatory markers and lipoprotein profile

**Fig. 2:**
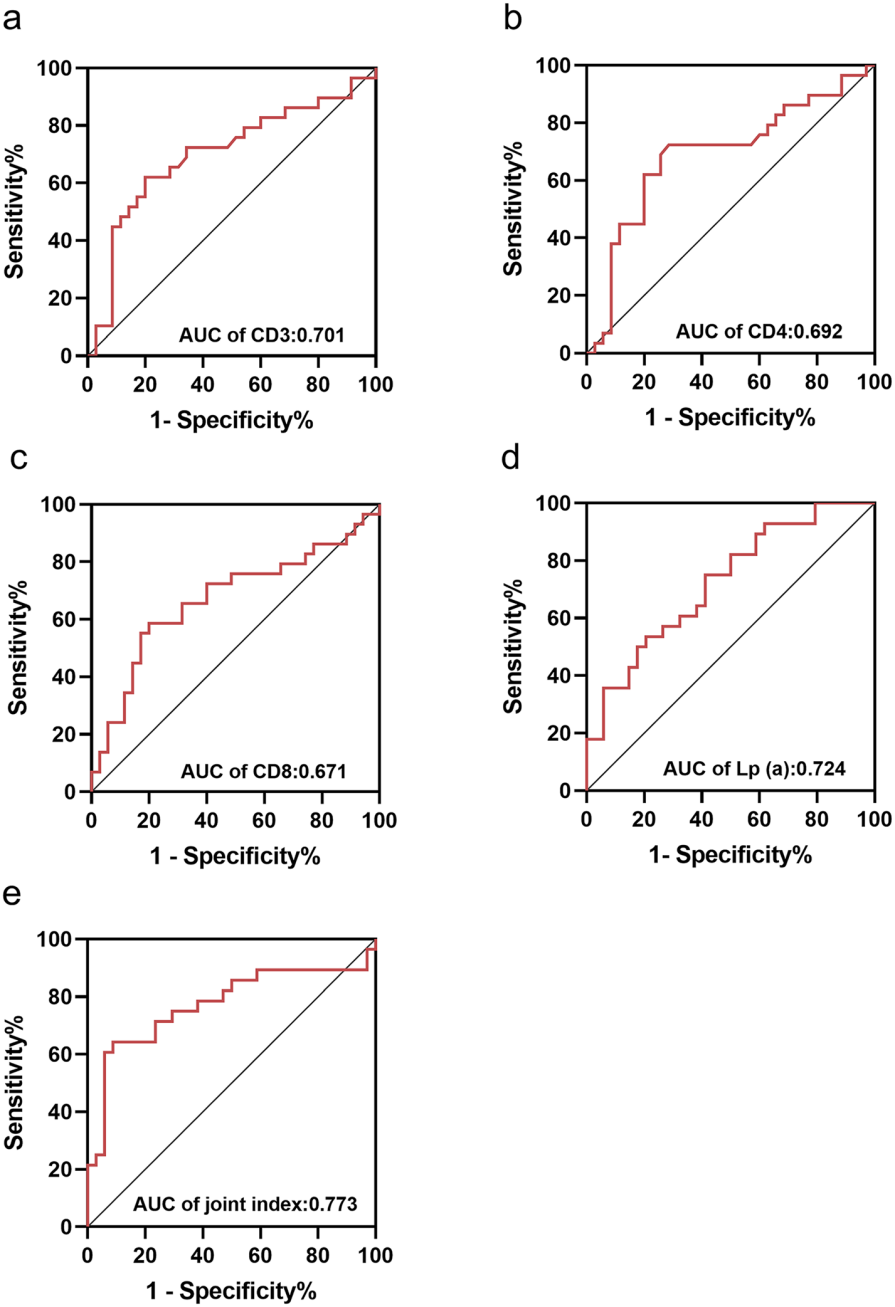
Area under the curves reflect the discriminating anhedonia in male patients with unipolar and bipolar depression. **a.** AUC value of the single CD3 was 0.701. **b.** AUC value of the single CD4 was 0.692. **c.** AUC value of the single CD8 was 0.671. **d.** AUC value of the Lp (a) was 0.724. **e.** AUC of the combinations of CD3, CD4 and CD8 and Lp (a) value was 0.773. Abbreviations: AUC, area under the curve; Lp (a), lipoprotein(a)

ROC curve analysis showed that the AUCs of CD3, CD4, CD8, and Lp (a) were 0.701, 0.692, 0.671, and 0.724, respectively (Fig. 2A-D). The highest AUC was obtained for the combination of all four biomarkers, with a specificity of 91.2% (Fig. 2E). The combination of CD3, CD4, CD8, and Lp (a) for anhedonia diagnosis had the strongest predictive value on logistic analysis. The cutoff values, sensitivity, specificity, AUCs, and *P*-values are shown in Table 4.

**Table 4:** Area under the curves reflect the discriminating anhedonia in male patients with unipolar and bipolar depression

Variables	Cutoff Point	Sensitivity (95%CI)	Specificity (95%CI)	AUC (95%CI)	*P*
CD3	1726.00	0.621 (0.440–0.773)	0.800 (0.641-0.900)	0.701 (0.565–0.836)	0.006**
CD4	944.00	0.724 (0.543–0.853)	0.714 (0.550–0.837)	0.692(0.555–0.828)	0.009**
CD8	742.00	0.586 (0.407–0.745)	0.800 (0.641-0.900)	0.671 (0.532–0.810)	0.019**
Lp (a)	68.00	0.750 (0.566–0.873)	0.588 (0.422–0.736)	0.724(0.600-0.849)	0.003**
Combination^a^	0.45	0.643 (0.458–0.793)	0.912 (0.770–0.970)	0.773 (0.642–0.901)	0.0002***

Abbreviation: Lp(a), lipoprotein(a)

* *P* < 0.05; ** *P* < 0.01; *** *P* < 0.001

^a^Combination diagnosis based on logistic model formula

## Discussion

To the best of our knowledge, the current study is one of the first to investigate immune-inflammatory markers and lipoprotein profiles in patients with and without anhedonia with unipolar and bipolar depression according to sex. Herein, we present three major findings. First, the anhedonia group only presented higher levels of CD3, CD4, and CD8 and lower levels of Lp (a) in male patients with unipolar and bipolar depression. Second, CD3, CD4, CD8, and Lp (a) levels were significantly associated with anhedonia in male participants with unipolar and bipolar depression. Third, the combination of CD3, CD4, CD8, and Lp (a) had the strongest predictive value for distinguishing anhedonia in male participants with unipolar and bipolar depression. Collectively, these findings suggest that CD3, CD4, CD8, and Lp (a) might be possible biomarkers of anhedonia in male patients with unipolar and bipolar depression.

We found elevated levels of CD3, CD4, and CD8 and lower levels of Lp (a) in the anhedonia group, which appears to be specific for male patients. These findings suggest that anhedonia in male patients with unipolar and bipolar depression may present specific immune-inflammatory markers and lipoprotein profiles, as well as more severe immune-inflammatory disturbances. Male and female individuals demonstrate distinct peripheral and central inflammatory and immune processes in both ex vivo and in vivo experiments [43]. Evidence from rodent studies has also found peripheral inflammatory changes in male rats after CUMS [23]. Relatively low Lp (a) in male patients with anhedonia suggests that Lp (a) may have protective effects against unipolar and bipolar depression. This is in line with the findings of Moreira et al. [44] reported that individuals with anhedonia had significantly decreased lipoprotein levels. Conversely, additional evidence has reported that female individuals may be more vulnerable to the depressogenic effects of neuroinflammation [43]. Female-specific alterations in inflammation, lipid levels, and depression have also been reported. These seemingly contradictory findings might be attributed to brain, gene expression, and behavioral characteristics, coping styles, life experiences, cultural expectations, and inherent biological differences throughout one’s lifespan [1, 9, 11, 32]. Another possible explanation is that male individuals may present significantly higher levels of immune-inflammatory markers (e.g., CD3, CD4, and CD8) [45], while female individuals may demonstrate relatively greater risk for inflammation-related depression [46]. Future longitudinal studies examining sex differences in unipolar and bipolar depression, immune-inflammatory markers and lipoprotein profiles, and anhedonia are needed to validate these findings. Collectively, both immune-inflammatory markers and lipoprotein profiles may contribute to potential sex differences in patients with anhedonia.

Furthermore, higher levels of CD3, CD4, and CD8 and lower levels of Lp (a) were significantly associated with greater anhedonia in male patients with unipolar and bipolar depression. These results further support the hypothesis of immune-related or inflammatory etiology of anhedonia in depression [47]. CD4 + T cells play a key role in the induction of T-lymphocyte proliferation, and CD8 + T cells perform immunosuppressive functions [48]. Significantly altered levels of CD4 and CD8 T cells suggests disturbed immune surveillance and inflammation [23]. Anhedonia symptoms in male CUMS rats are associated with immune inflammatory markers (CD3+, CD4+, and CD4+ /CD8 + ratio) [23]. Clinical studies have also reported that patients with anhedonia have elevated levels of proinflammatory cytokines (e.g., IL-6, IL-1β, and TNF-α) compared to patients without anhedonia in depression [49, 50] several lines of evidence support a role for inflammation in the pathophysiology, etiology, and treatment outcomes of anhedonia in depression [51]. Jha et. reported that elevated T cell cytokines were only associated with anhedonia severity in male patients [52]. Neuroinflammation can also affect lipid regulation in the central nervous system. Correspondingly, lipoproteins also regulate immune and inflammatory responses in humans [53]. Loas et al. also found an association between anhedonia and low lipid levels in patients with depression [54]. Lp (a) promotes the activation of T-helper-1 (Th1) and natural killer (NK) cells [55]. Collectively, immune-inflammatory markers and lipoproteins play a major role in the pathogenesis of anhedonia in depression, indicating that anti-inflammatory interventions that target male patients might be more effective for patients with anhedonia patients with unipolar and bipolar depression.

Notably, CD3, CD4, CD8, and Lp (a) had limited predictive value for anhedonia; however, the combination of CD3, CD4, CD8, and Lp (a) showed the strongest predictive value for distinguishing anhedonia in male patients with unipolar and bipolar depression. The neuroinflammatory responses in anhedonia involve inflammatory markers, and the lipoprotein profile is regulated by many factors that interact with each other [56, 57]. The combination of multiple biomarkers into a diagnostic panel could improve the accuracy of early identification [58]. Consistent with this, Zhou et al. constructed a joint index, including the CD4+/CD8 + T cell ratio, to discriminate depression severity [48]. Thus, a combination of different immune-inflammatory markers (namely, CD3, CD4, and CD8) and lipoprotein profile [Lp (a)] is more likely to reveal unique neuroinflammatory responses in anhedonia. In line with this, we suggest that CD3, CD4, CD8, and Lp (a) are potential biomarkers of anhedonia. This joint index was based on sex-specific biomarkers from peripheral blood, which could facilitate easier and more accurate anhedonia classification in unipolar and bipolar depression. This study may pave the way for precision medicine and personalized treatment options in patients with anhedonia.

The present study has some limitations. First, we did not compare immune-inflammatory markers and lipoprotein profiles between healthy controls and the study groups since each biomarker employed a clear reference range, as was used in these analyses. Second, given the cross-sectional design, we could not uncover the interrelationship among immune-inflammatory markers, lipoprotein profiles, and anhedonia in patients with unipolar and bipolar depression. Therefore, the findings presented herein should be interpreted with caution. Further research on these factors could provide insights into the molecular mechanisms underlying anhedonia in patients with unipolar and bipolar depression. Third, the relatively small sample size, particularly in subgroup analyses, limited our statistical power. The biomarkers identified in this study can be replicated in larger multicenter samples. Fourth, information on medication intake was not collected in the present dataset, and thus, we cannot rule out the effect of unmeasured confounding in our analysis. Future studies in medication naïve patients are needed to clarify these issues.

## Conclusions

This study adds to the literature by showing that CD3, CD4, and CD8 levels are elevated, while Lp (a) is downregulated in male patients with anhedonia. Our study suggests that both immune-inflammatory and lipoprotein profile alterations may contribute to sex differences in anhedonia in patients with unipolar and bipolar depression. Furthermore, the combination of CD3, CD4, CD8, and Lp (a) may be a promising biomarker associated with anhedonia in male patients with unipolar and bipolar depression. These findings provide valuable insights for understanding sex-specific biomarkers and molecular mechanisms of anhedonia in patients with unipolar and bipolar depression. If verified, this study has considerable implications in precision medicine.

### Electronic supplementary material

Below is the link to the electronic supplementary material.

**Supplementary Material 1**: Sex-specific immune-inflammatory markers and lipoprotein profile in patients with anhedonia with unipolar and bipolar depression

## Data Availability

The datasets used and/or analysed during the current study available from the corresponding author on reasonable request.

## List of abbreviations

AUC: area under the curve
CUMS: Chronic Unpredictable Mild Stress
HAMA: Hamilton Anxiety Rating Scale
HAMD: Hamilton Depression Scale
HDL: high-density lipoproteins
LDL: low-density lipoproteins
Lp (a): lipoprotein(a)
Or: Odds ratio
ROC: receiver operating characteristic
SCID-P: the Structured Clinical In-terview for DSM-IV-TR-Patient Edition
SHAPS: Snaith-Hamilton Pleasure Scale
TC: total cholesterol
TEPS: Temporal Experience of Pleasure Scale
TG: triglycerides
YMRS: Young Mania Rating Scale
