# Supplementary material for: Sex-specific immune-inflammatory markers and lipoprotein profile in patients with anhedonia with unipolar and bipolar depression
Source: BMC Psychiatry. 2023 Nov 27;23:879. doi: 10.1186/s12888-023-05378-4 (PMC10680275; doi:10.1186/s12888-023-05378-4)
Supplement: Supplementary file 1 — Supplementary Material 1: Sex-specific immune-inflammatory markers and lipoprotein profile in patients with anhedonia with unipolar and bipolar depression [file 12888_2023_5378_MOESM1_ESM.doc]

**Supplementary Information for**

**Sex-specific immune-inflammatory markers and lipoprotein profile in patients with anhedonia with unipolar and bipolar depression**

Shengjuan Lin ^1, 2†^, Rongxun Liu ^1, 2, 3†^, Zhongguo Zhang ^4^, Fengyi Liu ^5^, Shisen Qin ^5^, Yange Wei ^6*^, Fei Wang ^1, 2, 3*^

^1^ Early Intervention Unit, Department of Psychiatry, The Affiliated Brain Hospital of Nanjing Medical University, Nanjing, China

^2^ Functional Brain Imaging Institute, Nanjing Medical University, Nanjing, China.

^3^ School of Psychology, Xinxiang Medical University, Xinxiang, China

^4^ The Fourth People’s Hospital of Yancheng, Yancheng, China

^5^ School of Public Health, Xinxiang Medical University, Xinxiang, China

^6^ Department of Early Intervention, The Second Affiliated Hospital of Xinxiang Medical University, Henan Mental Hospital, Xinxiang, China

^†^ Shengjuan Lin and Rongxun Liu have contributed equally to this work.

***Correspondence:**

Fei Wang, Early intervention Unit, Department of Psychiatry. The Affiliated Brain Hospital of Nanjing Medical University, 264 Guangzhou Street, Nanjing, Jiangsu, 210029, China; Department of Psychology, Xinxiang Medical University, Xinxiang, China. Email: [fei.wang@yale.edu](mailto:fei.wang@yale.edu)

Yange Wei, Department of Early Intervention. The Second Affiliated Hospital of Xinxiang Medical University,

Henan Mental Hospital, Xinxiang, 453002, Henan, China. Email: weiyange@xxmu.edu.cn

Table S1. Demographic and clinical characteristics in patients with unipolar and bipolar depression grouped based on sex.

| Characteristic | Total patients (N = 227) | Male (N = 64) | Female (N = 163) | t | *P* |
| --- | --- | --- | --- | --- | --- |
| Age (years) | 15.57±2.08 | 15.59±1.92 | 15.56±2.15 | 0.094 | 0.925 |
| BMI (kg/m^2^) | 23.38±7.26 | 24.52±7.39 | 22.92±7.19 | 0.654 | 0.157 |
| HAMA total score | 23.28±7.96 | 21.98±8.64 | 23.78±7.65 | -1.525 | 0.129 |
| HAMD total score | 23.31±6.71 | 21.82±7.02 | 23.88±6.51 | -2.703 | 0.039* |
| YMRS total score | 9.31±6.25 | 9.35±6.02 | 9.30±6.36 | 0.061 | 0.952 |
| TEPS total score | 67.87±17.34 | 66.09±18.11 | 68.85±16.90 | -1.018 | 0.310 |
| TEPS ANT score | 36.70±9.57 | 37.06±10.23 | 36.54±9.31 | 0.362 | 0.718 |
| TEPS CON score | 28.35±8.71 | 29.03±8.90 | 28.07±8.64 | 0.734 | 0.465 |
| CD3 (μL) | 2006.32 ± 701.30 | 1948.88 ± 695.04 | 2028.87 ± 704.59 | -0.773 | 0.441 |
| CD4 (μL) | 844.57 ± 339.57 | 977.56 ± 357.55 | 1049.87 ± 361.15 | -1.361 | 0.175 |
| CD8 (μL) | 844.57 ± 339.57 | 843.69 ± 325.73 | 844.92 ± 345.84 | -0.025 | 0.980 |
| TC (mmol/L) | 4.08 ± 0.92 | 3.99 ± 0.97 | 4.11 ± 0.90 | -0.876 | 0.382 |
| TG (mmol/L) | 1.08 ± 0.65 | 1.22 ± 0.64 | 1.02 ± 0.65 | 2.042 | 0.042* |
| LDL (mmol/L) | 2.12 ± 0.66 | 2.13 ± 0.73 | 2.12 ± 0.64 | 0.096 | 0.924 |
| HDL (mmol/L) | 1.29 ± 0.30 | 1.18 ± 0.20 | 1.33 ± 0.32 | -3.426 | 0.001** |
| Lp (a) (mg/L) | 176.53 ± 229.57 | 146.06 ± 142.08 | 188.42 ± 255.11 | -1.562 | 0.120 |

Abbreviations: BMI, body mass index; HAMA, Hamilton Anxiety Rating Scale; HAMD-17, Hamilton Depression Rating Scale-17; YMRS, Young Mania Rating Scale; TEPS, Temporal Experience of Pleasure Scale; TEPS ANT, TEPS anticipatory anhedonia; TEPS CON, TEPS consummatory anhedonia. TC, total cholesterol; TG, triglycerides; LDL, low-density lipoproteins; HDL, high-density lipoproteins; Lp (a) , lipoprotein(a). * *P* < 0.05; ** *P* < 0.01; *** *P* < 0.001.

Table S2. Demographic and clinical characteristics in patients with unipolar and bipolar depression.

| Characteristic | Male (N = 64) | | | | Female (N = 163) | | | |
| --- | --- | --- | --- | --- | --- | --- | --- | --- |
|  | Unipolar depression  (N = 29) | Bipolar  depression  (N = 35） | t | *P* | Unipolar depression  (N = 79) | Bipolar depression  (N = 84） | t | *P* |
| Age (years) | 15.07±1.46 | 16.00±2.16 | -1.947 | 0.056 | 15.33±1.89 | 15.77±2.36 | -1.325 | 0.187 |
| BMI (kg/m^2^) | 24.58±7.35 | 24.46±7.54 | 0.057 | 0.955 | 22.56±7.86 | 23.26±6.48 | -0.580 | 0.563 |
| HAMA total score | 22.35±9.48 | 21.68±7.98 | 0.304 | 0.762 | 24.18±7.22 | 23.40±8.06 | 0.642 | 0.522 |
| HAMD total score | 22.14±7.67 | 21.56±6.54 | 0.324 | 0.747 | 24.05±5.86 | 23.72±7.11 | 0.319 | 0.750 |
| YMRS total score | 8.21±5.29 | 10.29±6.49 | -1.363 | 0.178 | 7.51±5.25 | 11.02±6.87 | -3.661 | < 0.001*** |
| TEPS total score | 65.45±19.85 | 66.63±16.80 | -0.258 | 0.798 | 64.36±16.31 | 73.54±16.36 | -3.038 | 0.003** |
| TEPS ANT score | 36.90±10.83 | 37.20±9.86 | -0.117 | 0.907 | 34.92±8.45 | 38.15±9.89 | -2.200 | 0.029* |
| TEPS CON score | 28.55±9.62 | 29.43±8.39 | -0.389 | 0.698 | 27.12±7.96 | 29.01±9.22 | -1.379 | 0.170 |
| CD3 (μL) | 1943.86±712.63 | 1953.03±690.55 | -0.052 | 0.959 | 2064.41±674.40 | 1995.45±734.31 | 0.623 | 0.534 |
| CD4 (μL) | 971.31±327.29 | 982.74±385.50 | -0.126 | 0.900 | 1065.72±349.11 | 1034.95±373.60 | 0.542 | 0.588 |
| CD8 (μL) | 842.62±365.76 | 844.57±293.97 | -0.024 | 0.981 | 864.18±323.58 | 826.81±366.56 | 0.688 | 0.492 |
| TC (mmol/L) | 4.20±1.08 | 3.81±0.85 | 1.566 | 0.123 | 4.03±0.77 | 4.20±1.00 | -1.174 | 0.242 |
| TG (mmol/L) | 1.30±0.68 | 1.15±0.59 | 0.928 | 0.357 | 0.95±0.54 | 1.09±0.75 | -1.364 | 0.175 |
| LDL (mmol/L) | 2.25±0.81 | 2.02±0.64 | 1.272 | 0.208 | 2.03±0.57 | 2.21±0.69 | -1.750 | 0.082 |
| HDL (mmol/L) | 1.22±0.19 | 1.14±0.21 | 1.672 | 0.100 | 1.33±0.27 | 1.33±0.36 | 0.097 | 0.923 |
| Lp (a) (mg/L) | 137.48±136.84 | 153.61±148.23 | -0.443 | 0.659 | 155.32±176.52 | 221.10±311.80 | -1.640 | 0.104 |

Abbreviations: BMI, body mass index; HAMA, Hamilton Anxiety Rating Scale; HAMD-17, Hamilton Depression Rating Scale-17; YMRS, Young Mania Rating Scale; TEPS, Temporal Experience of Pleasure Scale; TEPS ANT, TEPS anticipatory anhedonia; TEPS CON, TEPS consummatory anhedonia. TC, total cholesterol; TG, triglycerides; LDL, low-density lipoproteins; HDL, high-density lipoproteins; Lp (a) , lipoprotein(a). * *P* < 0.05; ** *P* < 0.01; *** *P* < 0.001.
